# The role of nesfatin-1 in kidney diseases

**DOI:** 10.1007/s00467-024-06569-1

**Published:** 2024-10-31

**Authors:** Marta Badeńska, Andrzej Badeński, Artur Janek, Maria Szczepańska

**Affiliations:** Department of Pediatrics, Faculty of Medical Sciences in Zabrze, Medical University of Silesia in Katowice, ul. 3 Maja 13-15, 41-800 Zabrze, Poland

**Keywords:** Nesfatin-1, Kidney, Chronic kidney disease, Acute kidney injury, Blood pressure, Renal cell carcinoma

## Abstract

Nesfatin-1 is a recently discovered protein with a pleiotropic function on various organs, including kidneys. This molecule presents antiapoptotic, antihyperglycemic, antioxidative, and anorectic features. Available data regarding the role of nesfatin-1 in kidney function and diseases focuses on chronic kidney disease, acute kidney injury, blood pressure, and renal cell carcinoma. Various studies have shown that the levels of nesfatin-1 were increased in patients with diabetic kidney disease (DKD); therefore, it was suggested that nesfatin-1 might act as an early DKD marker. Furthermore, the potential protective function of nesfatin-1 against inflammation, oxidative stress, fibrosis, and apoptosis in kidney tissues was described in several studies. Alternatively, as reported in the literature, a positive correlation between blood pressure elevation and nesfatin-1 levels was noted. Moreover, nesfatin-1 might exert influence on renal cell carcinoma progression and invasion of cancerous cells. Nesfatin-1 shows considerable potential for acting as a prognostic marker or a defensive factor for kidney diseases; however, further investigation, especially in the pediatric population, is still required.

**Graphical abstract:**

**Figure:**
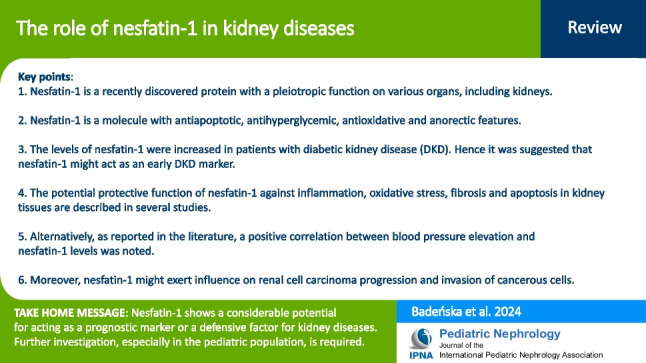
A higher resolution version of the Graphical abstract is available as Supplementary information

**Supplementary Information:**

The online version contains supplementary material available at 10.1007/s00467-024-06569-1.

## Introduction

Nesfatin-1 was discovered in 2006 when Oh-I et al*.* conducted a study on the regulation of food intake. The source of nesfatin-1 is nucleobindin-2 (NUCB2). Previous research into the structure of NUCB2, a protein encoded by the *NUCB2* gene, led to describing a number of potential cleavage sites in the molecule [1]. Cleaving NUCB2 by prohormone convertase 1/3 (PC1/3) resulted in production of nesfatin-1, -2, and -3 [2–4]. Nesfatin-1, a protein formed by 82 amino acids, includes three domains — N-terminal (N23), middle (M30), and C-terminal (C29) [1]. Its expression was primarily described in hypothalamic nuclei in rats [1]. Nesfatin-1 can cross the blood–brain barrier; however, peripheral production was documented mainly in gastric and intestinal mucosa, adipose and muscle tissue, and pancreatic β-cells [4–6], whereas the main portion is produced by gastric mucosa and subcutaneous adipocytes [4, 7].

In the literature, food intake regulation is reported to be the main nesfatin-1 feature. Studies have proved a strong negative correlation between nesfatin-1 and neuropeptide Y, a widely acknowledged orexigenic peptide. Both proteins are believed to exert a suppressing effect on each other, which further results in a regulation of vacuity and repletion [2, 5, 8, 9]. Moreover, nesfatin-1 is believed to block the dopaminergic neurons, therefore minimizing food intake by decreasing dopamine release [5]. There are several results of anorexigenic nesfatin-1 function. Firstly, the reduction of food intake generates weight loss and reduced gain of adipose tissue [6, 10, 11]. Secondly, the correlation between fluctuations of endogenic nesfatin-1 levels and obesity is reported in several studies. In overweight and obese patients, the levels of endogenic nesfatin-1 were elevated [3, 9, 12, 13]. On the contrary, its levels were decreased in fasting individuals, as well as after bariatric surgery [8, 9, 14, 15].

Another widely reported feature of nesfatin-1 is glucose homeostasis modulation. Primarily, nesfatin-1 levels have been reported to be lower in diabetic patients in comparison with healthy individuals [4, 15–19]. Surprisingly, in cases of impaired glucose tolerance and prediabetes, nesfatin-1 serum concentrations were elevated compared to normoglycemic patients [20, 21]. Several studies have concluded that nesfatin-1 exerts a peripheral, glucose-dependent effect on insulin production increase; therefore, an intravenous injection of this protein resulted in serum glucose level lowering in patients with hyperglycemia [3, 7–9, 16, 19].

Various studies have presented nesfatin-1’s influence on stress. Research using animal models showed that stress could modulate the expression of NUCB2/nesfatin-1 in the nervous system in goldfish [22], as well as rats [23, 24]. On the other hand, central nervous system (CNS) injection of nesfatin-1 resulted in ACTH and corticosterone and serum level increases in rats [25] and cortisol in goldfish [22]. Moreover, Bonnet et al. reported an increase in nesfatin-1 expression in CNS due to peripheral inflammation, suggesting its impact on endotoxemic anorexia in parallel [26].

Nesfatin-1 has different functions in various organs, which confirms its pleiotropic nature. Estimating its role in kidneys remains under research, especially in pediatric populations (Table 1). Chung et al. [27] conducted a study comparing nesfatin-1 expression in adult, fetus, and neonatal mice. The levels of nesfatin-1 in the kidneys were significantly higher in adult rodents in comparison with neonatal and fetus. However, the authors claim that more research is required to explain the role of this protein in development and its function in different organs. Sun et al. [28] evaluated the expression of nesfatin-1 in the mouse fetus and found that the level of the protein fluctuated in different stages of fetal development in several organs, including the kidney, lung, and heart. Therefore, it is possible that nesfatin-1 might have an impact on growth, maturation, and function of those organs.

**Table 1:** A summary of the role of nesfatin-1 in kidney-related diseases

Kidney-related diseases	Nesfatin-1 role
Acute kidney injury (AKI) — in vitro and animal models	• Reducing inflammation • Reducing cell viability and nuclear swelling • Reducing oxidative stress • Minimizing tubular apoptosis
Chronic kidney disease (CKD) — animal model	• Decreasing blood urea nitrogen, creatinine, lactate dehydrogenase, uric acid, and urine protein • Reducing cell fibrosis • Minimizing inflammatory response
Diabetic kidney disease (DKD)	• Correlating with urinary albumin excretion and hemoglobin A1c — possible early DKD marker • Reducing oxidative stress • Reducing cell fibrosis
Blood pressure (BP) alterations	• Elevating BP • Increasing in obese-related hypertension in children — possible marker for hypertension in obesity • Increasing sodium and water reabsorption
Renal cell carcinoma (RCC)	• Correlating with advanced stages of cancer and increased invasion — possible marker of RCC progression

## The role of nesfatin-1 in the kidneys

### Acute kidney injury

The current knowledge of pathogenesis of acute kidney injury (AKI) is still scarce. The plausible causal agents most reported in the literature are oxidative stress, increased inflammatory response, and apoptosis. Srashti et al. constructed a hypothesis that nesfatin-1, being an antiapoptotic protein, may be correlated with progression of kidney lesions in AKI. The research entailed assessment of nesfatin-1 expression in rodents and in vitro models of AKI. Furthermore, the authors evaluated the impact of nesfatin-1 on kidney cells incubated with nephrotoxic agent (doxorubicin). The outcome revealed a significant decrease of nesfatin-1 levels in AKI-induced models. Moreover, a possible protective feature of the protein was also discovered, since a considerable reduction of inflammatory markers, such as tumor necrosis factor alpha (TNF-α) and interleukin 1β (IL-1β), was noted in cells incubated with doxorubicin as well as cell viability and nuclear swelling being reduced [29].

Among other pathologies, the ischemia/reperfusion (I/R) injury, frequently occurring due to clinical procedures such as vascular surgery, partial nephrectomy, or kidney transplantation, may quite often lead to AKI. Jiang et al. conducted a study evaluating the impact of intraperitoneal nesfatin-1 on kidney I/R injury in a murine model. According to the collected data, the authors suggested that nesfatin-1 might have an inhibitory effect on kidney I/R injury through oxidative stress reduction and minimizing tubular apoptosis. Leading mechanisms described in the study included reducing lipid peroxidation and inhibiting the infiltration of activated leucocytes into the kidney cells [30].

### Chronic kidney disease

Tezcan et al. aimed to evaluate the influence of nesfatin-1 on the damage of kidney cells observed in chronic kidney disease (CKD). An experimental rat model, created by inducing unilateral ureteral obstruction (UUO), was designed to imitate pathological lesions in CKD, such as tubular injury, interstitial inflammation, and tubulointerstitial fibrosis. The authors concluded that nesfatin-1 exerts anti-inflammatory and anti-fibrotic effects on kidney tissues through the expression of nesfatin-1 receptor in tubular epithelial cells and vascular endothelial and immune cells. The study suggested the role of nesfatin-1 in decreasing the blood urea nitrogen (BUN) level, as well as inhibiting the expression of alpha smooth muscle actin (α-SMA) — the indicator of fibrosis. Moreover, experimental treatment with nesfatin-1 minimized the inflammatory response by reducing infiltration of immune cells into the kidney. According to the study, nesfatin-1 could reverse renal tubulointerstitial fibrosis to some extent; therefore, the authors suggested that it could improve the prognosis for patients with kidney diseases [31].

A comparable analysis by Lahane et al. aimed to assess potential reparative properties of nesfatin-1 in an animal with CKD using adenine. Features of CKD, such as apoptosis, fibrosis, inflammation, and biochemical disorders, were described in the adenine-induced mice. These pathological processes tended to reverse after the nesfatin-1 application. The study presented a role for nesfatin-1 in improving serum creatinine, BUN, lactate dehydrogenase (LDH), uric acid, and urine protein, as well as reduction of inflammatory cytokines activity, transforming growth factor beta (TGF-β), α-SMA, and collagen IV [32].

Saldanha et al. conducted research that assessed nesfatin-1 in adult patients treated with hemodialysis (HD). Such an innovative clinical study might have led to significant observations as HD patients are at greatest risk of cachexia due to HD and the advanced stage of CKD itself. However, the outcome exposed no significant differences in nesfatin-1 concentrations between HD patients and control group. Moreover, the authors suggested that malnutrition among CKD patients is probably not caused by alterations in the nesfatin-1 pathway. Still, the research confirmed the anorexigenic features of this protein, since its levels were negatively correlated with markers of nutrition and protein intake. Furthermore, it was believed that the most significant factor causing nesfatin-1 fluctuations was percentage of body fat (% BF). In the literature, there are several reports describing inflammation in association with nesfatin-1 expression in the brain stem. Therefore, the authors highlighted the phenomenon of lack of correlation between nesfatin-1 and TNF-α or C-reactive protein (CRP), elevated among patients with CKD [33, 34].

Further studies regarding CKD focused on scrutinizing levels of circulating nesfatin-1 in patients with diabetic kidney disease (DKD). Irannejad et al. advanced a hypothesis of a reasonable impact of nesfatin-1 in DKD pathogenesis, since this protein serves to regulate glycemia. The results of the research demonstrated significantly higher nesfatin-1 levels in patients with diabetes mellitus (DM) and microalbuminuria compared to the control group — diabetic patients without kidney complications. Moreover, significant correlations between nesfatin-1 levels and urinary albumin excretion (UAE) as well as hemoglobin A1c (HbA1c) were highlighted; therefore, this protein was presented as a potential marker for early DKD diagnosis [35].

An additional study regarding DKD, conducted by Korani et al., aimed to assess nesfatin-1 levels in various stages of DKD in patients with DM type 2. The outcome revealed increased plasma nesfatin-1 levels in patients with macroalbuminuria. The authors observed a significant negative correlation between nesfatin-1 levels and estimated glomerular filtration rate (eGFR). Moreover, nesfatin-1 levels were correlated with severity of DKD. The protein was also presented as a potential marker for early proteinuria and DKD development among patients with DM [36].

Lahane et al. conducted research demonstrating a protective role of nesfatin-1 in high glucose-induced and oxidative stress-related renal epithelial cell injury. The authors suggested that oxidative stress caused by high glucose levels might be one of the main mechanisms of kidney damage. Therefore, factors minimizing oxidative stress may serve as remedies for kidney diseases. Firstly, a significant decrease of nesfatin-1 levels was observed in kidney cells exposed to oxidative stress as well as correlated with increase in inflammatory cytokines expression. Using nesfatin-1 as a medical remedy resulted in reduction of oxidative distress and increase of endogenic antioxidant enzymes. Moreover, a regeneration of cells along with reduction of TGF-β — an indicator of fibrosis, was observed. The authors highlighted that the underlying molecular mechanisms for describing nesfatin-1 protective functions are yet to be explored [37].

### Blood pressure

Renal sympathetic nerve activity, altering the pressure-natriuresis relationship, may influence blood pressure (BP) through modifications in kidney hemodynamics, urinary sodium excretion, and the renin-angiotensin system [38]. Tanida et al. highlighted the impact of intracerebroventricular (ICV) injections of nesfatin-1 on central metabolic regulation [1, 39, 40] and raised the hypothesis that this protein may exert influence on sympathetic nerve activity. Therefore, a rat model was constructed to evaluate cardiovascular response to nesfatin-1 ICV injection. The authors described a prominent elevation of BP through sympathetic nerve activation, most likely triggered by nesfatin-1 acting via the melanocortin system. Therefore, it was assumed that ICV application of nesfatin-1 may influence the cardiovascular homeostasis through renal sympathetic nerve activity modulation [41]. A subsequent study by Tanida et al. described further mechanisms of nesfatin-1 regulation of the autonomic nervous system. The outcome revealed that the protein injected centrally activated the extracellular signal-regulated kinase (ERK) in a rat model, consequently influencing kidney sympathetic nerves and BP [42].

Comparable research was performed by Osaki et al., who aimed to assess the effect of peripheral nesfatin-1 injection on BP in rats. Subcutaneous injections of the protein resulted in a significant elevation of BP values, while heart rate was not affected. Moreover, the authors showed that the nesfatin-1-induced increase of BP was associated with beta-adrenergic receptor involvement. The authors suggested that the main mechanism of BP fluctuations upon nesfatin-1 injection was linked with a direct influence on vascular smooth muscle contraction [43].

Obesity-related hypertension, increasing in prevalence among patients of all ages, is believed to be caused by several factors, such as the renin–angiotensin–aldosterone system activation, the abovementioned stimulation of the sympathetic nerves, and hyperinsulinemia and alterations in functions of certain adipokines. Güneş et al. compared levels of nesfatin-1 in groups of children with obesity and hypertension. According to the study, a significant increase of nesfatin-1 level was observed in obese children with hypertension compared to obese, normotensive individuals. Moreover, an increase of nesfatin-1 level was noted in correlation with older age. The authors highlighted that nesfatin-1 might serve as the predictor of hypertension in obesity, since its levels were independently related to BP elevation [44].

In addition, several studies mentioned the co-expression of nesfatin-1 with epithelial sodium channels (ENaC) in renal collecting ducts, resulting in modifications in sodium and water reabsorption in the kidney and consequently decreased sodium urine excretion. Such mechanisms may result in plasma volume increase and, along with the abovementioned influence on sympathetic nerve and beta-receptor activity, lead to a significant increase in blood pressure [45–47].

### Renal cell carcinoma 

Research regarding pathogenesis of renal cell carcinoma (RCC) has shown a major impact of obesity and metabolic disorders in the development of the disease. Since nesfatin-1’s fundamental function is food intake regulation, Chinapayan et al. presented a systematic review describing the role of nesfatin-1 in development of RCC and assessing its potential to predict the progression of the illness. The outcome revealed a significant positive correlation between the level of adipokine and progression of RCC. Moreover, higher levels of nesfatin-1 were associated with advanced stages of kidney cancer and increased invasion of cancerous cells [48]. In addition, nesfatin-1 and NUCB2 knockout in the kidney cancer cell line resulted in inhibition of proliferation and metastasizing [49, 50]. Nesfatin-1 precursor, NUCB2, was also assessed in terms of prediction of renal clear cell carcinoma (ccRCC), the most frequent histological variant of RCC. The findings suggested a strong correlation of NUCB2 with tumor growth and metastases, as well as a higher mortality. The authors presented NUCB2 as a potential early marker of ccRCC; however, further studies on larger cohorts are required [51].

## Conclusions

**Fig. 1:**
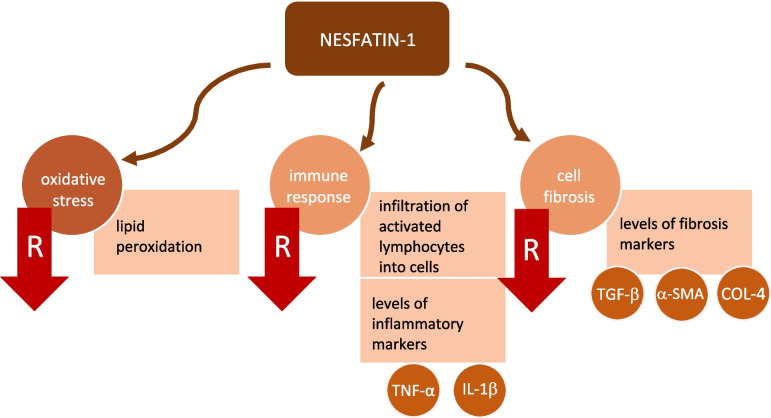
Selected nesfatin-1 functions influencing processes involved in kidney disorders. R, reduction; TNF-α, tumor necrosis factor alpha; IL-1β, interleukin-1 beta; TGF-β, transforming growth factor beta; α-SMA, alpha-smooth muscle actin; COL-4, collagen IV

Nesfatin-1 is a molecule with a pleiotropic function on various organs. According to the literature, its impact on kidney disorders is multifactorial. Its expression was noted due to inflammation; furthermore, nesfatin-1 was believed to minimize inflammatory response, apoptosis, and oxidative stress, consequently serving as a protective agent in fibrosis and acute kidney damage. On the other hand, several studies reported its higher levels in RCC, particularly among advanced stages of the disease with poorer prognosis. Moreover, the influence of nesfatin-1 on BP is most probably induced by diverse mechanisms, altogether leading to elevated BP values. In the literature, knowledge of nesfatin-1 in kidney diseases focuses on adult patients; therefore, its role in kidney disorders in the pediatric population should be explored in future studies (Fig. 1).

Nesfatin-1 is a recently discovered protein that shows a considerable potential for acting as a prognostic marker or a defensive factor for kidney diseases. Further investigation is required.

## Supplementary Information

Below is the link to the electronic supplementary material.

Graphical abstract (PPTX 72 KB)
