# Supplementary material for: The role of nesfatin-1 in kidney diseases
Source: Pediatr Nephrol. 2024 Oct 31;40(4):901–7. doi: 10.1007/s00467-024-06569-1 (PMC11885357; doi:10.1007/s00467-024-06569-1)
Supplement: Supplementary file 1 — Graphical abstract (PPTX 72 KB) [file 467_2024_6569_MOESM1_ESM.pptx]

## Slide 1
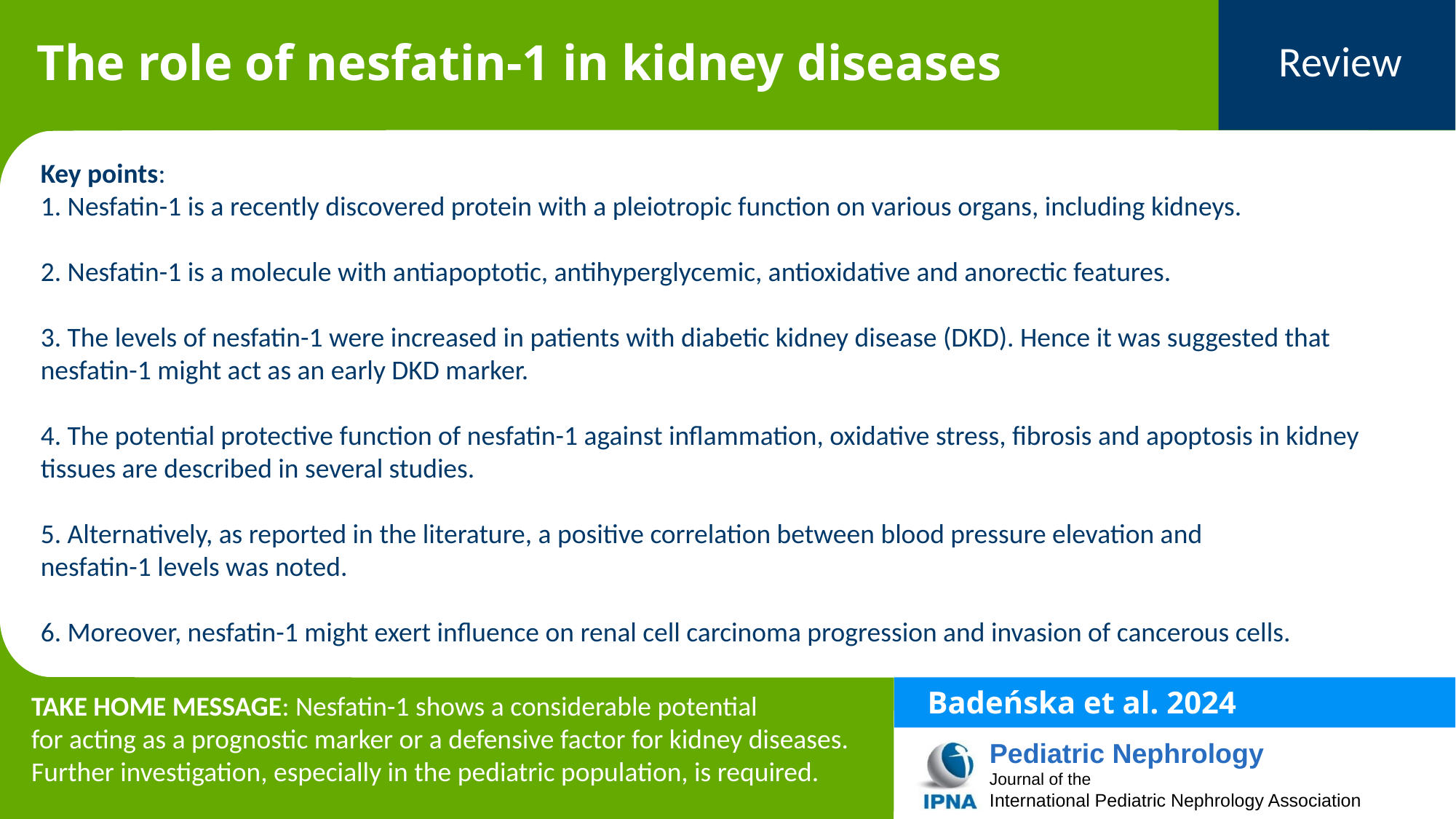

The role of nesfatin-1 in kidney diseases
Key points:
1. Nesfatin-1 is a recently discovered protein with a pleiotropic function on various organs, including kidneys.
2. Nesfatin-1 is a molecule with antiapoptotic, antihyperglycemic, antioxidative and anorectic features.
3. The levels of nesfatin-1 were increased in patients with diabetic kidney disease (DKD). Hence it was suggested that nesfatin-1 might act as an early DKD marker.
4. The potential protective function of nesfatin-1 against inflammation, oxidative stress, fibrosis and apoptosis in kidney tissues are described in several studies.
5. Alternatively, as reported in the literature, a positive correlation between blood pressure elevation and
nesfatin-1 levels was noted.
6. Moreover, nesfatin-1 might exert influence on renal cell carcinoma progression and invasion of cancerous cells.
Badeńska et al. 2024
TAKE HOME MESSAGE: Nesfatin-1 shows a considerable potential
for acting as a prognostic marker or a defensive factor for kidney diseases. Further investigation, especially in the pediatric population, is required.
